# Direct Electrochemical Generation of Catalytically Competent Oxyferryl Species of Classes I and P Dye Decolorizing Peroxidases

**DOI:** 10.3390/ijms222212532

**Published:** 2021-11-20

**Authors:** Magalí F. Scocozza, Lígia O. Martins, Daniel H. Murgida

**Affiliations:** 1 Departamento de Química Inorgánica, Analítica y Química Física, Facultad de Ciencias Exactas y Naturales, Universidad de Buenos Aires, Buenos Aires C1428EGA, Argentina; magaliscocozza@gmail.com; 2 Instituto de Química Física de los Materiales, Medio Ambiente y Energía (INQUIMAE), CONICET-Universidad de Buenos Aires, Buenos Aires C1428EGA, Argentina; 3 Instituto de Tecnologia Química e Biológica António Xavier, Universidade Nova de Lisboa, 2780-157 Oeiras, Portugal; lmartins@itqb.unl.pt

**Keywords:** dye-decolorizing peroxidases, compound I, compound II, Raman spectroscopy, spectroelectrochemistry

## Abstract

This work introduces a novel way to obtain catalytically competent oxyferryl species for two different dye-decolorizing peroxidases (DyPs) in the absence of H_2_O_2_ or any other peroxide by simply applying a reductive electrochemical potential under aerobic conditions. UV-vis and resonance Raman spectroscopies show that this method yields long-lived compounds II and I for the DyPs from *Bacillus subtilis* (BsDyP; Class I) and *Pseudomonas putida* (PpDyP; Class P), respectively. Both electrochemically generated high valent intermediates are able to oxidize ABTS at both acidic and alkaline pH. Interestingly, the electrocatalytic efficiencies obtained at pH 7.6 are very similar to the values recorded for regular catalytic ABTS/H_2_O_2_ assays at the optimal pH of the enzymes, ca. 3.7. These findings pave the way for the design of DyP-based electrocatalytic reactors operable in an extended pH range without the need of harmful reagents such as H_2_O_2_.

## 1. Introduction

Dye decolorizing peroxidases (DyPs) are a relatively novel family of heme peroxidases that uses hydrogen peroxide as an electron source for catalyzing the oxidation, and in some cases the hydrolysis, of a wide variety of substrates. This includes lignin, phenolic and non-phenolic lignin units, synthetic dyes, aromatic sulfides and even metals ions such as iron and manganese [1,2,3,4,5]. While DyPs were first identified and purified in 1999 [6,7], it took almost a decade to recognize them as an independent peroxidase family with unique features [8]. The research on DyPs has been gaining momentum since then, providing valuable insights into the structural and mechanistic aspects of these enzymes and leading to the identification and implementation of potential and actual industrial applications, respectively [9,10].

To date, DyP-type peroxidase sequences have been detected in fungi, mycetozoa, bacteria and archaea [11]. They present diverse molecular functions and physiological roles and can therefore be classified into distinct subfamilies. In prokaryotes, DyPs which belong to class I (former type A) present a Tat signal, which indicates that they function outside the cytoplasm, as in the case of the *Bacillus subtilis* enzyme (BsDyP) [12,13]. Classes P and V (former type B and C, respectively) comprise cytoplasmic enzymes involved in intracellular metabolism. A typical class P enzyme is the DyP from *Pseudomonas putida* (PpDyP) [12,14]. DyPs from fungal origins also belong to class V, formerly classified as type D [15,16].

**Scheme 1:**
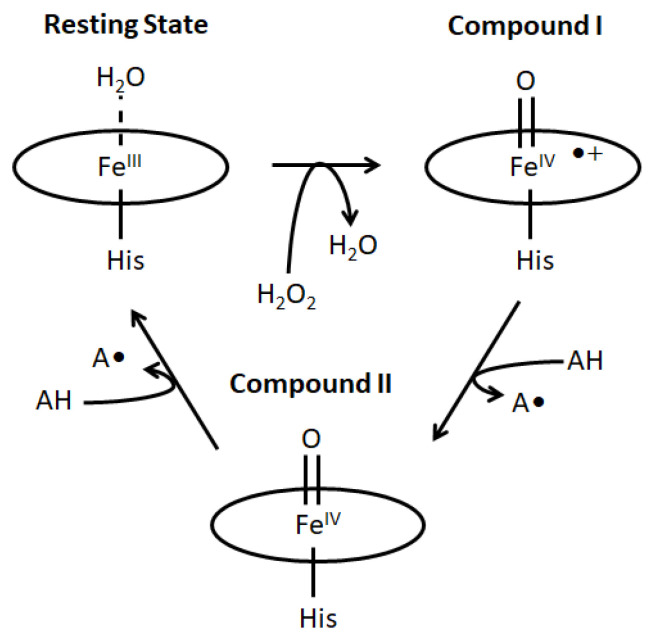
Minimal catalytic mechanism proposed for general peroxidases [22], AH stands for one equivalent of substrate that is oxidized by the enzyme to A•.

The catalytic mechanism of general peroxidases is shown in Scheme 1. Compound I has been identified for DyPs from different classes at optimum pH [13,14,17,18]; however, generation of compound II by addition of solely H_2_O_2_, i.e., in the absence of reducing substrates, has only been detected at neutral pH [19]. According to Shrestha et al. [20], these results suggest that DyPs might follow a different catalytic mechanism in which compound I returns to the resting state directly through two-electron reduction without forming compound II. On the other hand, Martins and coworkers demonstrated the formation of both compounds I and II throughout the catalytic cycles of BsDyP and PpDyP at pH 3.8 and 8.5, respectively, in the presence of H_2_O_2_ and a reducing substrate and provided evidence that the mechanism comprises two one-electron reduction steps [13,21].

Aspartic acid and arginine are conserved in the H_2_O_2_-binding site of most DyPs, in contrast to general peroxidases, which present histidine and arginine at this site. The distal aspartate is responsible for the deprotonation of the bound H_2_O_2_, which requires previous deprotonation of this residue [23]. For that reason, optimal catalytic efficiencies for DyPs are achieved at fairly acidic pH, usually between 3 and 4, and are virtually inactive at neutral and alkaline pH [12,15]. This last feature may represent a serious drawback for some applications. Another crucial characteristic of these enzymes is that the binding site(s) for reducing substrates are different from the H_2_O_2_ binding site, in cavities that give direct access to the heme, and close to aromatic residues located at the protein surface that can establish efficient long-range electron transfer pathways to the heme [24,25]. Indeed, numerous studies have identified surface tryptophan and tyrosine residues as radical sites able to perform substrate oxidation [26,27,28,29,30].

Here, we present an electrochemical and spectroscopic study of two different DyP enzymes: the class I BsDyP and the class P PpDyP. We show that these enzymes form long-lived oxyferryl species by simple application of a reductive electrode potential under aerobic conditions. Interestingly, the applied potential does not reduce the heme iron but serves to activate molecular oxygen. To the best of our knowledge, this is the first report of generation of oxyferryl species in peroxidases without the need for H_2_O_2_ or any other typical reagents such as m-chloroperoxybenzoic acid or p-nitroperoxybenzoic acid and using only molecular oxygen instead. Specifically, BsDyP stabilizes compound II, while PpDyP stabilizes compound I. This novel way to generate oxyferryl species enables efficient catalytic oxidation of the prototypical dye 2,2′-Azino-bis(3-ethylbenzothiazoline-6-sulfonic acid) diammonium salt (ABTS) both at acidic and alkaline pH without the need for harmful chemicals such as H_2_O_2_, thus paving the way for the design of electrocatalytic enzymatic reactors for different applications.

## 2. Results

### 2.1. Generation and Spectroscopic Characterization of BsDyP and PpDyP High-Valent Catalytic Intermediates

**Figure 1:**
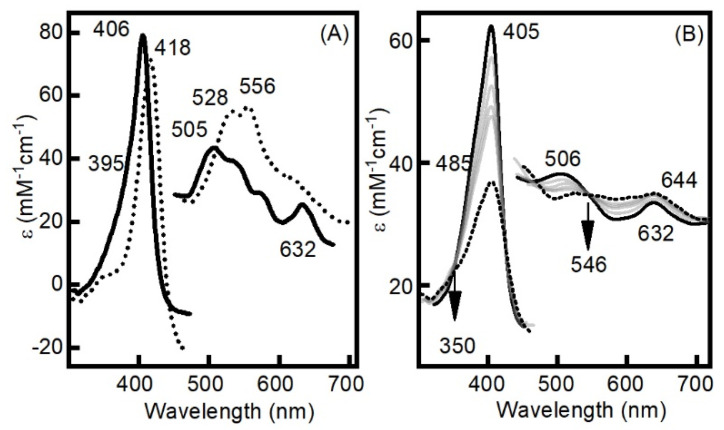
UV-vis spectra of (**A**) BsDyP, at open circuit potential (thick solid line) and at −0.190 V potential (dashed line); (**B**) PpDyP at open circuit potential (thick solid line), changes within first two minutes at −0.190 V potential (grey solid line) and final −0.190 V product (dashed line).

**Figure 2:**
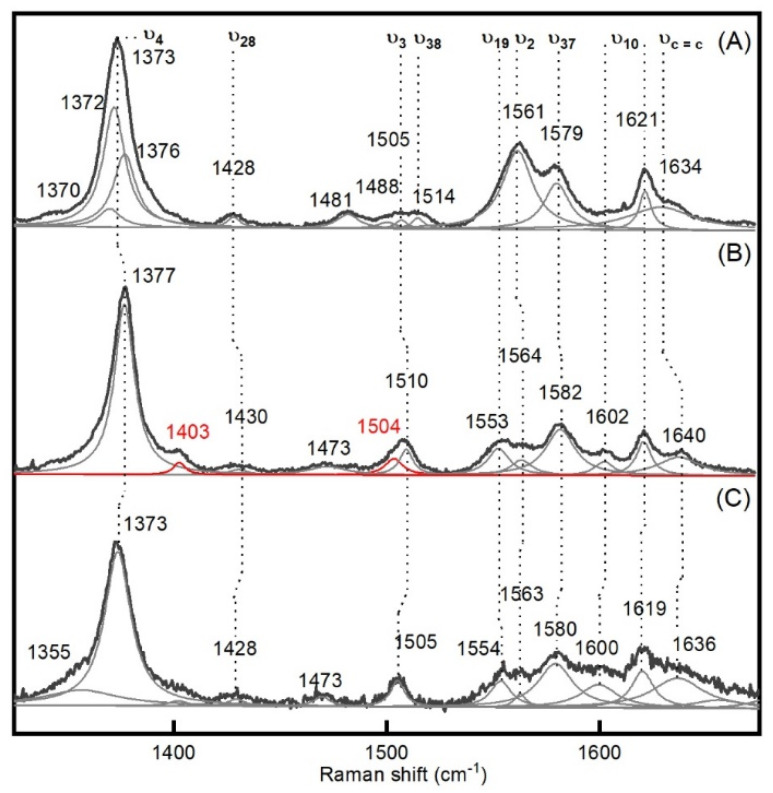
RR spectra of BsDyP recorded with Soret band excitation at pH 7.6 and 298 k under aerobic conditions. (**A**) Resting enzyme, measured at open circuit potential. (**B**) Compound II, measured at −0.190 V applied potential. (**C**) Compound I, obtained after addition of ferrocyanide to the product of aerobic electrolysis at −0.190 V shown in panel (**B**). Black lines are experimental spectra. Gray lines are the result of spectral deconvolution and correspond to Lorentzian RR bands of the heme. Bands and labels in red are assigned to tyrosyl radical stretchings.

The resting states of the ferric as-purified BsDyP and PpDyP enzymes were characterized by UV-vis absorption and resonance Raman (RR) spectroscopies at pH 7.6, yielding the same spectral features recently reported [31]. Briefly, the absorption spectrum of BsDyP exhibits a Soret maximum at 406 nm with a small shoulder at 395 nm, Q-bands at 505 and 540 nm and a charge transfer band at 632 nm (Figure 1A). The RR core size marker bands show a heterogenous axial coordination of the BsDyP heme iron, consisting of: (i) a six coordinated low spin population (6cLS) represented by the bands at 1376 (ν_4_), 1505 (ν_3_) and 1579 (ν_37_) cm^−1^; (ii) a six coordinated high spin (6cHS) population with bands at 1372 (ν_4_), 1481 (ν_3_), 1514 (ν_38_) and 1561 (ν_2_) cm^−1^; and (iii) a five coordinated high spin (5cHS) population with bands at 1370 (ν_4_) and 1488 (ν_3_) (Figure 2A) [31,32].

**Figure 3:**
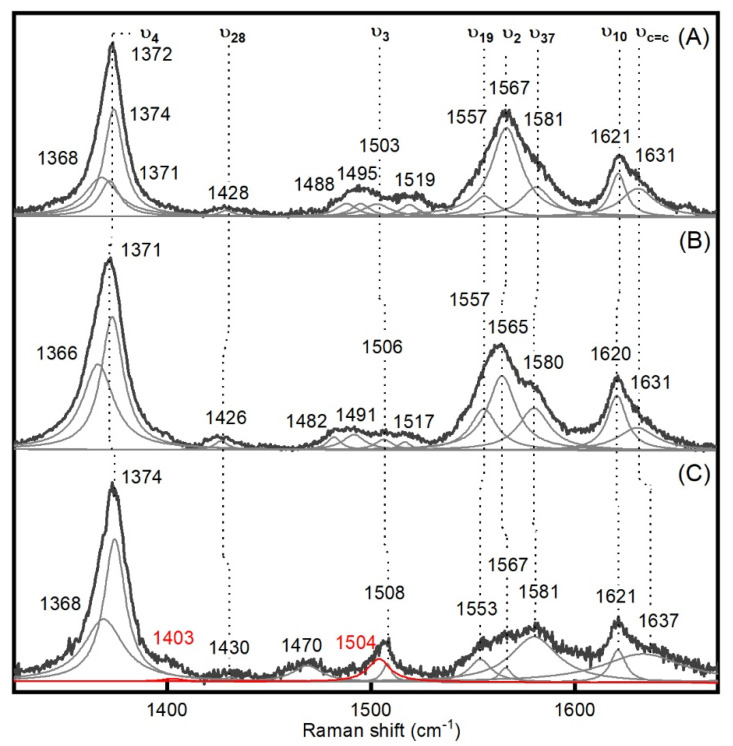
RR spectra of PpDyP recorded with Soret band excitation at pH 7.6 and 298 k under aerobic conditions. (**A**) Resting enzyme, measured at open circuit potential. (**B**) Compound I, measured at −0.190 V applied potential. (**C**) Compound II, obtained after addition of ferricyanide to the product of aerobic electrolysis at −0.190 V shown in panel (**B**). Black lines are experimental spectra. Gray lines are the result of spectral deconvolution and correspond to Lorentzian RR bands of the heme. Bands and labels in red are assigned to tyrosyl radical stretchings.

PpDyP, on the other hand, presents the Soret band at 405 nm with a shoulder at 385 nm, the Q-band at 506 nm and the charge transfer band at 632 nm (Figure 1B). The RR spectrum reveals three different populations regarding the heme axial coordination and spin: (i) a 6cHS fraction with bands at 1368 (ν_4_), 1488 (ν_3_), 1518 (ν_38_) 1557 (ν_19_) cm^−1^; (ii) a 5cHs form with bands at 1371 (ν_4_), 1495 (ν_3_), 1567 (ν_2_) and 1581 (ν_37_) cm^−1^; and (iii) a five-coordinated quantum mechanically mixed-spin (5cQS) population, characterized by bands at 1374 (ν_4_), 1503 (ν_3_) and 1525 (ν_38_) cm^−1^ (Figure 3A). This latter spin species has also been observed in class III plant peroxidases and catalase peroxidases [32,33].

Unlike the type B DyP from *Klebsiella pneumoniae* and some other peroxidases [34,35], attempts to reduce the resting BsDyP and PpDyP enzymes by either direct or mediated electrochemistry under Ar atmosphere were unsuccessful as no cyclic voltammetry (CV) signals could be detected, and furthermore, application of electrode potentials as negative as −0.190 V in a three electrode spectroelectrochemical cell resulted in no changes of the UV-vis and RR spectra. In sharp contrast, application of the same reductive potential under air atmosphere, i.e., in the presence of oxygen, results in strong cathodic CV peaks (Figure S1) and distinct changes of the UV-vis and RR spectra for both enzymes, yielding identical results in the presence and absence of a cocktail of redox mediators (see Section 3.1). 

Application of −0.190 V to BsDyP under aerobic conditions leads to a hypochromic red shifting of the Soret and Q absorption bands to 418 nm and 528 and 556 nm, respectively (Figure 1A). The conversion of the resting state to this form is completed within 3 minutes upon potential application and presents three isosbestic points at 414, 458 and 521 nm (Figure S2). The final spectrum is similar to those reported for the chemically generated compound II oxyferryl species for this and other peroxidases [12,13,18]. Formation of this species leads to upshifts of the ν_4_, ν_28,_ ν_3_, ν_2_, ν_37_ and ν_C=C_ bands of the RR spectrum (Table 1 and Figure 2B), which are consistent with the formation of a 6cLS [Fe(IV)=O] species. In addition to these signals, the RR spectrum of the electrochemically treated BsDyP exhibits two bands at 1504 and 1403 cm^−1^ that cannot be attributed to core-size bands of the heme. Similarly to the case of the chemically generated compound II of bovine liver catalase [36], the most likely assignments for these bands are CC and CO stretchings of a tyrosyl radical [37]. Based on these results, we assign the product of the aerobic electroreductive treatment of BsDyP as a compound II tyrosyl radical species, (Fe(IV)=O)Tyr•.

In the case of the PpDyP enzyme, aerobic electroreductive treatment at −0.190 V leads to a significant drop, albeit with no shifts, of the Soret band, and upshifts of the Q and charge transfer bands, with isosbestic points at 350 and 546 nm (Figure 1B) that result in a greenish coloration. These changes, which are completed within two minutes, are consistent with the formation of a compound I oxoferryl species [12,14,18], in sharp contrast with the BsDyP enzyme. Deconvolution of the RR spectrum of this species reveals a minor fraction of the Fe(II) enzyme with ν_4_ = 1366 cm^−1^, along with a main 6cLS component consistent with compound I formation (Figure 3B and Table 1) [38]. RR spectroscopy is sensitive to the coordination number, spin and redox state of the heme iron, as well as to the oxidation of the porphyrin macrocycle. The two alternative [Fe(IV)=O] species, i.e., compounds I and II, are both 6cLS and have the same iron oxidation number; the main difference is the π-cation radical character of the porphyrin in compound I, which has a predominantly ^2^A_2u_ ground state configuration. This results in downshifts (Δν_ox_) of most skeletal stretching vibrations when compound I is chemically oxidized to compound II [38]. A comparison of the RR spectra recorded for the electrochemically generated oxyferryl species of BsDyP and PpDyP (Figure 2 and Figure 3 and Table 1) indeed shows this trend, in agreement with their assignments as compounds II and I, respectively.

Interestingly, the addition of ferrocyanide to the electrochemically generated compound I from PpDyP leads to upshifts of the RR marker bands, which are consistent with its reduction to compound II (Figure 3C and Table 1). The RR spectrum of the obtained PpDyP compound II resembles that of BsDyP, both regarding the position of the porphyrin marker bands and the presence of additional bands at 1403 and 1504 cm^−1^ attributable to the formation of a tyrosyl radical (Figure 2 and Figure 3), thus leading to a (Fe(IV)=O)Tyr• species.

Likewise, the addition of ferricyanide to the electrochemically generated BsDyP compound II results in an RR spectrum that is characteristic of a 6cLS form, albeit with downshifted heme marker bands with respect to the initial compound II and in the disappearance of the tyrosyl radical bands (Figure 2C). There is also a minor fraction of the Fe(II) enzyme with ν_4_ = 1355 cm^−1^. These results are consistent with the oxidation of compound II to compound I, as previously described for other peroxidases and catalases [39].

Altogether, these results show that the application of reductive electrode potentials to BsDyP and PpDyP under aerobic conditions leads to the generation of compounds II and I species, respectively, which are spectroscopically identical to the intermediates generated by addition of H_2_O_2_ to the same enzymes [12]. In agreement with these findings, a previous study suggests that class I DyPs preferentially stabilize the compound II intermediate, while class P enzymes stabilize compound I in their reactions with H_2_O_2_ at pH 7.6 [18].

The mechanism of formation of the oxyferryl species remains unknown, and its elucidation requires extensive studies that are beyond the scope of the present work. What is clear is that the formation of these species involves atmospheric molecular oxygen dissolved in the enzyme solution and requires application of reductive potentials. Based on these observations, we speculate that the process may involve electroreduction of aromatic residues at the protein surface, which in turn react with O_2_ to generate ROS, such as superoxide. This species may reduce the enzyme to Fe(II), as long established for peroxidases [40]. The reduced enzyme might bind O_2_ and generate compounds I and II following a mechanism similar to cytochrome P450’s [41]. This process, however, has to be fast as no Fe(II) intermediate species were detected in the time scale of the spectroscopic experiments. Alternatively, superoxide disproportionation in water leads to H_2_O_2_, which can eventually react with the enzyme. The formation of H_2_O_2_ by superoxide disproportionation, however, is less favorable in alkaline media [42].

**Figure 4:**
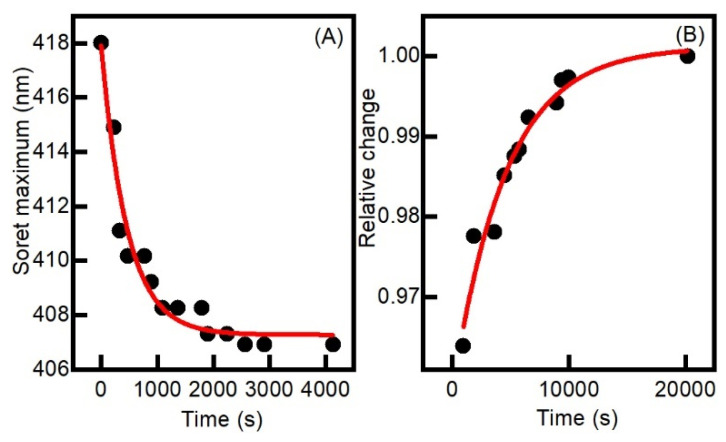
Decay of BsDyP compound II (**A**) and PpDyP compound I (**B**) to the respective resting states, as monitored by UV-vis absorption spectroscopy immediately after opening the electrochemical cell circuit. The ordinate axis represent the position and relative intensity of the Soret band, respectively.

The electrochemical generation of oxyferryl species is reversible; upon opening the electrochemical cell circuit, both enzymes return to their resting states as monitored by UV-vis absorption, with half-life times at 298 K of 4.6 and 52 min for BsDyP compound II and PpDyP compound I, respectively (Figure 4). When the reductive potential is maintained under aerobic conditions for periods longer than ca. 45 min. a gradual loss of absorbance is observed, probably due to heme bleaching by accumulated ROS, albeit with no qualitative spectral changes.

### 2.2. Aerobic versus Anaerobic Redox Titrations of BsDyP and PpDyP

**Figure 5:**
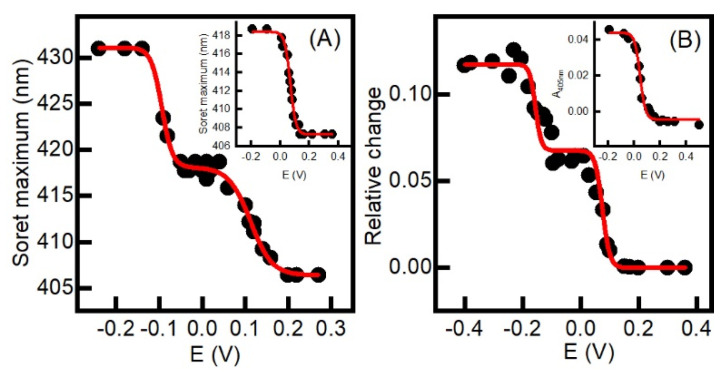
Redox titrations of BsDyP (**A**) and PpDyP (**B**) with sodium dithionite under aerobic conditions and monitored by UV-vis spectroscopy. The potentials correspond to the open circuit potential of the 3-electrode OTTLE cell and are reported versus NHE. The ordinate axis in panel (**A**) represents the shift of the Soret band from 406 nm to 418 nm for the formation of compound II, and from 418 nm to 431 nm for the full reduction. The ordinate axis in panel (**B**) are expressed in terms of relative change for practical purposes as the first transition, which corresponds to the formation of compound I, does not produce a shift of the Soret band but rather an intensity drop, while the second transition, which corresponds to the full reduction, implies a shift of the Soret band from 405 to 431 nm. The insets show the direct aerobic electrochemical titrations of the enzymes without addition of redox reagents. Potentials in this latter case correspond to closed-circuit values against NHE.

The anaerobic redox titration of BsDyP has been reported before [43], yielding broad transitions with reduction potentials for the Fe(III)/Fe(II) redox couple, E°′ (Fe^3+^/Fe^2+^), between −40 and −80 mV. In this case, the reduction is characterized by the upshift of the Soret absorption maximum from 406 to 430 nm. Direct electrochemical reduction under aerobic conditions, in contrast, leads to the formation of (Fe(IV)=O)Tyr• compound II, which has the Soret band at 418 nm, and no reaction is detected under anaerobic conditions (vide supra). Monitoring the electrochemical conversion from the resting state to compound II by either UV-vis or RR spectroscopies (Figures S3A and S4) leads to sigmoid transitions that fit Nernst equation for one electron oxidation with an apparent oxidation potential $E{{}^{\circ}}^{\prime }\left({\mathrm{Fe}}^{3+}/\mathrm{CII}\right)$ = 0.071 ± 0.002 V (Figure 5A inset). 

In contrast, aerobic titrations of BsDyP with sodium dithionite monitored by UV-vis and RR and recording the open circuit potential of the three electrode OTTLE cell (Figures S6 and S7) lead to two consecutive transitions of one and two electrons, respectively (Figure 5A). The first transition corresponds to the formation of the 418 nm species assigned as compound II and exhibits an apparent redox potential $E{{}^{\circ}}^{\prime }\left({\mathrm{Fe}}^{3+}/\mathrm{CII}\right)$ = 0.112 ± 0.004 V, i.e., very close, within experimental error, to the value obtained by direct electrochemical generation of this species. The second transition corresponds to the reduction of compound II to Fe(II), as determined by the characteristic upshift of the Soret band to 430 nm and the downshift of the RR marker bands, including ν_4_ = 1355 cm^−1^ (Figures S6 and S7). The apparent potential of this reduction is $E{{}^{\circ}}^{\prime }\left(\mathrm{CII}/{\mathrm{Fe}}^{2+}\right)$ = −0.094 ± 0.003 V.

The detailed mechanism of the reactions is unknown, but based solely on the oxidation number of the iron, one can establish the following thermodynamic relationship:

(1) $$E{{}^{\circ}}^{\prime }\left({\mathrm{Fe}}^{3+}/{\mathrm{Fe}}^{2+}\right)=E{{}^{\circ}}^{\prime }\left({\mathrm{Fe}}^{3+}/\mathrm{CII}\right)+2E{{}^{\circ}}^{\prime }\left(\mathrm{CII}/{\mathrm{Fe}}^{2+}\right)$$

Replacing in Equation (1) the values of $E{{}^{\circ}}^{\prime }\left({\mathrm{Fe}}^{3+}/\mathrm{CII}\right)$ and $E{{}^{\circ}}^{\prime }\left(\mathrm{CII}/{\mathrm{Fe}}^{2+}\right)$ obtained from the aerobic titrations yield $E{{}^{\circ}}^{\prime }\left({\mathrm{Fe}}^{3+}/{\mathrm{Fe}}^{2+}\right)$ = −75 mV, i.e., very close to the values obtained through anaerobic titrations.

Likewise, the aerobic titration of PpDyP with sodium dithionite leads to two consecutive transitions involving three and two electrons, respectively (Figure 5B). The first transition corresponds to the formation of compound I, which does not produce a shift of the Soret band but rather an intensity drop. The second one corresponds to the reduction of compound I to Fe(II), which produces an upshift of the Soret maximum to 431 nm and the shift of characteristic RR marker bands, such as ν_4_ = 1353 cm^−1^ (Figures S8 and S9). Because the two transitions have different effects on the absorption spectra, the ordinate axes in Figure 5B are represented in terms of relative changes. The apparent redox potentials for these two processes are $E{{}^{\circ}}^{\prime }\left({\mathrm{Fe}}^{3+}/\mathrm{CI}\right)$ = 0.075 ± 0.002 V and $E{{}^{\circ}}^{\prime }\left(\mathrm{CI}/{\mathrm{Fe}}^{2+}\right)$ = −0.156 ± 0.006 V. On the other hand, UV-vis and RR monitoring of the direct aerobic electrochemical conversion of PpDyP resting state to compound I (Figures S3B and S5) leads to a sigmoid transition with apparent $E{{}^{\circ}}^{\prime }\left({\mathrm{Fe}}^{3+}/\mathrm{CI}\right)\;$= 0.047 ± 0.003 V (Figure 5B inset), which, considering the errors involved in the two different methodologies is relatively close to the value obtained by dithionite titration. Previous studies on PpDyP show a reduction potential for the Fe(III)/Fe(II) couple around −0.260 V [14].

As for BsDyP, the aerobic and anaerobic redox titrations of PpDyP can be related through the following thermodynamic relationship:

(2) $$E{{}^{\circ}}^{\prime }\left({\mathrm{Fe}}^{3+}/{\mathrm{Fe}}^{2+}\right)=2\;E{{}^{\circ}}^{\prime }\left({\mathrm{Fe}}^{3+}/\mathrm{CI}\right)+3E{{}^{\circ}}^{\prime }\left(\mathrm{CI}/{\mathrm{Fe}}^{2+}\right)$$

Introducing in Equation (2) the values of $E{{}^{\circ}}^{\prime }\left({\mathrm{Fe}}^{3+}/\mathrm{CI}\right)$ and $E{{}^{\circ}}^{\prime }\left(\mathrm{CI}/{\mathrm{Fe}}^{2+}\right)$ obtained from the aerobic titrations, we obtain $E{{}^{\circ}}^{\prime }\left({\mathrm{Fe}}^{3+}/{\mathrm{Fe}}^{2+}\right)$ = −0.318 V, which is close to the value of −0.260 V reported previously for anaerobic titrations [14].

### 2.3. The Electrochemically Generated Oxyferryl Species Are Catalytic 

The catalytic activity of the electrochemically generated BsDyP and PpDyP oxoferryl species at pH 3.7, 7.6, and 8.5 were evaluated using ABTS as substrate, without addition of H_2_O_2_. In these experiments, the OTTLE cell containing the enzyme and variable concentrations of substrate is poised at −0.190 V under aerobic conditions to generate a steady-state concentration of the oxyferryl species. UV-vis absorption spectroscopy monitoring of the reaction shows that both BsDyP and PpDyP are indeed able to efficiently oxidize ABTS in the absence of H_2_O_2_ in a wide pH range, specifically at alkaline pH at which these enzymes are inactive towards ABTS/H_2_O_2_ (Figures S10, S11, and S13) [12]. These results reinforce the idea that optimal pH of these enzymes at acidic conditions is probably determined by the first step of the reaction where an unprotonated Asp residue is fundamental. If this step is by-passed, the enzymes work fine at neutral to alkaline pHs.

**Figure 6:**
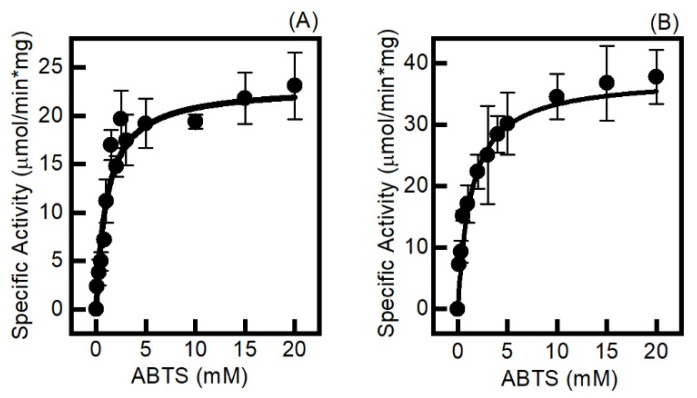
Steady-state kinetic analysis of the aerobic electrocatalytic oxidation of ABTS at pH 7.6, 298 K and a poised potential of −0.190 V for BsDyP (**A**) and PpDyP (**B**).

Michaelis–Menten curves obtained with this approach are shown in Figure 6, and the relevant parameters are summarized in Table 2. For the sake of comparison, data corresponding to standard ABTS/H_2_O_2_ tests of these enzymes at their optimal pH = 3.7/4.0 are also included. A key difference in these experiments with variable pH is that while PpDyP always forms compound I at poised −0.190 V regardless of the pH, BsDyP stabilizes compound I at pH 3.7 and compound II in alkaline medium, as verified by UV-vis absorption. Concomitantly, the *K_M_* value of BsDyP is significantly lower at pH 3.7 than at alkaline pH, thus suggesting a correlation between the nature of the stable oxyferryl species and *K_M_*. On the other hand, electrocatalytic efficiencies, *k_cat_/K_M_*_,_ of BsDyP measured at pH 3.7 and 7.6 are relatively similar, and close to the efficiency reported for H_2_O_2_/ABTS assays at pH 3.7 (Table 2) [44]. The electrocatalytic efficiency, however, drops by about two orders of magnitude at pH 8.5, partially due to a significantly lower *k_cat_*. Previous work has demonstrated that the reactivity of compound II decreases with increasing pH [39], which might explain the lower *k_cat_* of BsDyP at pH 8.5. 

In sharp contrast, the electrocatalytic efficiency of PpDyP is pH-independent in the entire range investigated and is very similar to the value obtained for the H_2_O_2_/ABTS assays at pH 4 (Table 2). 

Noteworthy, the *k_cat_* values obtained for the electrocatalytic activity of both enzymes appear to show a bell-shaped dependence with pH similar to that observed previously for H_2_O_2_/ABTS assays [12,13,14], except that for the electrochemically activated reactions, the maxima are upshifted by about 3 to 4 pH units.

As for other heme peroxidases [45,46,47], a recent crystallographic and docking study of BsDyP shows that bulky substrates preferentially bind to either the γ-edge site close to the heme propionate or to surface-exposed Tyr/Trp residues, from where electrons can be transferred to the heme via long-range electron transfer pathways [48]. It is very likely that the binding affinity of the tested substrate for the different sites is modulated by pH in a fashion that is not necessarily the same for both enzymes. On the other hand, recent studies on other DyPs have shown that the substrate–compound I interaction is the rate limiting step [20]. This suggests that the better overall electrocatalytic efficiency of PpDyP relative to BsDyP may be related to the fact that the first stabilizes compound I in the entire pH range, while the second stabilizes compound II at pH above 4 [20]. One should note, however, that for BsDyP and PpDyP, compound II reduction to the resting state has been shown to be rate limiting [13,21], albeit under conditions where H_2_O_2_ is utilized as electron source.

Certainly, further mechanistic work is required to establish the basis for the differences observed between electrochemically activated and H_2_O_2_ activated catalysis, as well as the intrinsic differences between the two enzymes and their pH dependencies. In any case, what is truly remarkable is the fact that both enzymes are amenable to H_2_O_2_-free electrochemical activation at alkaline pH and, therefore, are suitable candidates for their utilization in electrocatalytic enzymatic reactors. 

The fact that at pH 7.6 both enzymes are virtually inactive towards ABTS/H_2_O_2_ but present high electrocatalytic efficiencies may be attributed to the participation of a distal aspartate in the deprotonation of H_2_O_2_, which requires acidic pH [23]. This requisite is removed in the H_2_O_2_-free aerobic electrochemical activation of the enzymes.

## 3. Materials and Methods

### 3.1. Chemicals and Buffer Solutions

The buffer in all experiments was 20 mM TRIS-HCl at pH 7.6, containing 200 mM KCl. The following cocktail of redox mediators at different concentrations (2 µM, 5 µM, 10 µM) in buffer solution was used in the specified experiments: sodium 1,2-naphthoquinone-4-sulfonate (180 mV), trimethylhydroquinone (115 mV), methylene blue (11 mV), potassium indigotetrasulfonate (−30 mV), potassium indigotrisulfonate (−70 mV), 2-hydroxy-1,4-naphthoquinone (−152 mV), neutral red (−325 mV) and methyl viologen (−440 mV). All chemicals including buffer, redox mediators, sodium dithionite, potassium ferrocyanide, potassium ferricyanide, 2,2′-Azino-bis(3-ethylbenzothiazoline-6-sulfonic acid) diammonium salt (ABTS) and redox mediators were purchased from Sigma-Aldrich de Argentina SA, Buenos Aires, Argentina. ABTS solutions for catalytic activity measurements were prepared in the same buffer used for all other experiments. All chemicals were of highest purity grade available. All experiments were conducted with type II water (R > 18 MΩ). 

### 3.2. Construction, Overproduction and Purification of BsDyP and PpDyP

Plasmids pRC-1 containing PpDyP sequence and pVB4 containing BsDyP sequence with deletion of signal peptide were introduced into the host expression strain *E. coli* Tuner in which the target genes were expressed under the control of the T7lac promoter. Cell growth, disruption and protein purification were undertaken as previously described [12]. The protein concentration was determined by the Bradford assay with bovine serum albumin as standard. Purified enzymes were stored at −20 °C until use. The heme content was determined by the pyridine ferrohemochrome method using the extinction coefficient of pyridine hemochromes (R) minus the extinction coefficient of pyridine hemichromes (O) (ε(R − O) at 556 nm (28.32 mM^−1^ cm^−1^)) [49].

### 3.3. UV-Vis and Resonance Raman Experiments

Absorption spectra were acquired with an Evolution Array spectrophotometer from Thermo Scientific, Waltham, USA, using 1 mm quartz cuvettes.

Resonance Raman (RR) spectra were recorded in a Raman microscope (Dilor XY; f = 800 mm from Horiba Jobin Yvon, Lille, France) equipped with CCD detection and 1800 L/mm grating. Increments per data point were typically 0.4 cm^−1^, and accumulation times were ca. 10–30 s. Spectra were acquired using Soret band excitation (406 nm; TopMode-HP-406 diode laser Toptica Photonics AG, Munich; 3.5 mW at sample). Spectral calibration was done using chemical standards (silicon and 4-acetamidophenol).

After background subtraction, RR spectra were subjected to component analysis as described by Döpner et al. [50].

### 3.4. Spectroelectrochemical Experiments

All UV-vis experiments were carried out in a homemade 1 mm optical path length optical thin-layer electrochemistry (OTTLE, BAS Inc., Tokyo, Japan) cell with a three-electrode configuration: a gold grid working electrode, a Pt wire as counter electrode and Ag/AgCl (3.5 M KCl) reference electrode. The reference electrode was calibrated against a saturated calomel (HgCl) electrode before each set of measurements. All potentials reported in this work are referred to the standard hydrogen electrode. Potentials were applied across the OTTLE cell with either a teq3 (Sobral, Buenos Aires, Argentina) or PAR263A (Ametek Inc., Oak Ridge, TN, USA) potentiostat. A constant temperature was maintained by an alpha RA8 circulating water bath (Lauda; Lauda-Königshofen, Germany) and the temperature within the OTTLE cell was measured with thermocouple (Fluke 51 II; Fluke, Everett, WA, USA).

Spectroelectrochemical titrations were performed using 100 µL samples containing 30 µM heme protein in 20 mM TRIS-HCl buffer, pH 7.6 and 200 mM KCl.

For oxygen-free experiments, the OTTLE cell was flushed with argon gas. 

RR experiments with applied potential were carried out in a 1 cm optical path length quartz cuvette equipped with three electrodes similar to those used in UV-vis experiments, and magnetic stirring was maintained throughout the spectroscopic measurement to prevent laser induced degradation.

### 3.5. Steady State Kinetic Assays

Apparent steady-state kinetic constants (*k_cat_* and *K_M_*) for ABTS oxidation were obtained following its oxidation at 420 nm (ε_420nm_ = 36,000 M^−1^ cm^−1^) at different concentrations of substrate: from 0.1 mM to 20 mM. Data points were fitted using Michaelis–Menten equation.

### 3.6. Cyclic Voltammetry

Electrochemical experiments were performed with a Gamry REF600 electrochemical workstation (Gamry instruments Inc., Warminster, PA, USA) using a cell equipped with an edge plane pyrolytic graphite electrode (EPPGE; Allum Corp., Orlando, FL, USA), a Pt wire as counter electrode and Ag/AgCl (3.5 M KCl) reference electrode. EPPGE electrodes were polished with 1 µm diamond paste, cleansed with water and dried under an argon stream. The correct state of polished EPPGE was confirmed by Raman spectroscopy as described by Ray et al. [51] The temperature was controlled by a circulating water bath, and the temperature within the electrochemical cell was measured with a thermocouple (Fluke 51 II, Fluke, Everett, WA, USA).

## 4. Conclusions

The aerobic electrochemical treatment of the BsDyP and PpDyP enzymes at alkaline pH leads to the formation of intermediate species with lifetimes in the range of minutes that, according to their RR and UV-vis characterization, can be assigned as compounds II and I, respectively. The apparent oxidation potentials for the formation of these species are $E{{}^{\circ}}^{\prime }{\left({\mathrm{Fe}}^{3+}/\mathrm{CII}\right)}_{\mathrm{BsDyP}}$ = 75 ± 2 mV and $E{{}^{\circ}}^{\prime }{\left({\mathrm{Fe}}^{3+}/\mathrm{CI}\right)}_{\mathrm{PpDyP}}\;$= 47 ± 3 mV. The electrochemically generated compounds I and II can be interconverted by addition of [Fe(CN)_6_]^4−^/[Fe(CN)_6_]^3−^, further confirming their assignment.

The formation of these oxyferryl species is fast and quantitative, takes place without addition of H_2_O_2_ or any other peroxide and can be carried out at both acidic and alkaline pH, while these enzymes are inactive at alkaline pH when using H_2_O_2_ as the electron acceptor. The mechanisms of the reactions are unknown but may involve the electrochemical generation of tyrosyl radicals at the protein surfaces and subsequent reaction with molecular oxygen to yield superoxide, which in turn can reduce the enzymes enabling O_2_ binding or disproportionate to generate H_2_O_2_. The preferential stabilization of one or another oxyferryl species by these enzymes is in agreement with previous observations for classes I and P DyPs [18]. In the case of BsDyP, only compound II was detected at pH above 4, although transient formation of compound I cannot be discarded.

The stabilization and RR characterization of PpDyP compound I deserves a special mention. It is well established that lasers used for recording RR spectra usually lead to photoreduction or degradation of compound I, which imposes complex strategies for recording these spectra [38,52]. This is not the case for PpDyP compound I, whose RR spectrum could be measured under rather standard conditions without observing laser-induced degradation.

Of utmost importance is that the electrochemically generated oxyferryl forms of BsDyP and PpDyP are able to oxidize ABTS with high catalytic efficiencies over a broad pH range. At pH 7.6, the electrocatalytic efficiencies are similar to those reported for regular ABTS/H_2_O_2_ assays performed at the optimal pH of the enzymes, ca. 3.7. These findings pave the way for the design of DyP-based electrocatalytic reactors operable in an extended pH range without the need of corrosive and pollutant reagents such as H_2_O_2_.

## Figures and Tables

**Table 1:** Resonance Raman and UV-vis absorption marker bands for the resting state, compound I and compound II of the BsDyP and PpDyP enzymes. RR band positions are expressed in cm^−1^ and UV-vis in nm. Δν_ox_ refers to the difference in positions between compound I minus compound II.

Enzyme	Mode	Resting State	Compound I	Compound II	Δν_ox_
BsDyP	ν_4_	1373	1373	1377	−4
	ν_28_	1428	1428	1430	−2
	ν_3_	1505	1505	1510	−5
	ν_19_	-	1554	1553	+1
	ν_2_	1561	1563	1564	−1
	ν_37_	1579	1580	1582	−2
	ν_10_	-	1600	1602	−2
		1621	1619	1621	−2
	ν_C=C_	1634	1636	1640	−4
	UV-vis bands				
		406, 505, 540, 632	406, 524, 565	418, 528, 556	
PpDyP	ν_4_	1372	1374	1375	−1
	ν_28_	1428	1426	1430	−4
	ν_3_	1503	1506	1508	−2
	ν_19_	1557	1557	1553	+3
	ν_2_	1567	1565	1567	−2
	ν_37_	1581	1580	1581	−1
	ν_10_	1621	1620	1621	−1
	ν_C=C_	1631	1631	1637	−6
	UV-vis bands				
		405, 506, 632	405, 530, 562, 644	419, 528, 557	

**Table 2:** Apparent steady-state catalytic parameters of BsDyP and PpDyP for ABTS oxidation performed under two different conditions: (i) using H_2_O_2_ as electron source and (ii) in the absence of H_2_O_2_ at an applied potential of −0.190 V and aerobic atmosphere. All measurements were done at 298 K.

		pH	*k_cat_* (s^−1^)	*K_M_* (mM)	*k_cat_/K_M_* (s^−1^ M^−1^)
BsDyP	^1^ H_2_O_2_	3.7	15 ± 1	0.420 ± 0.030	3.6 × 10^4^
	Electrochemical	3.7	13 ± 1	0.141 ± 0.040	9.2 × 10^4^
		7.6	18 ± 1	1.155 ± 0.100	1.6 × 10^4^
		8.5	1.1 ± 0.1	2.334 ± 0.060	4.7 × 10^2^
PpDyP	^2^ H_2_O_2_	4	17 ± 1	1.349 ± 0.115	1.3 × 10^4^
	Electrochemical	3.7	4.2 ± 0.4	0.094 ± 0.016	4.5 × 10^4^
		7.6	19 ± 2	1.343 ± 0.190	1.4 × 10^4^
		8.5	3.2 ± 0.2	0.070 ± 0.010	4.6 × 10^4^

^1^ Taken from Rodrigues et al. [44]. ^2^ This work. See Figure S12.

## Supplementary Materials

The following are available online at https://www.mdpi.com/article/10.3390/ijms222212532/s1.
