# Supplementary material for: Direct Electrochemical Generation of Catalytically Competent Oxyferryl Species of Classes I and P Dye Decolorizing Peroxidases
Source: Int J Mol Sci. 2021 Nov 20;22(22):12532. doi: 10.3390/ijms222212532 (PMC8653965; doi:10.3390/ijms222212532)
Supplement: Supplementary file 1 [file ijms-22-12532-s001.zip › ijms-1441008 supplementary.pdf]

Supplementary information

Direct Electrochemical Generation of Catalytically Competent Oxyferryl Species of Classes I and P Dye Decolorizing Peroxidases

Magalí F. Scocozza[1,2], Lígia O. Martins[3] and Daniel H. Murgida[1,2]*

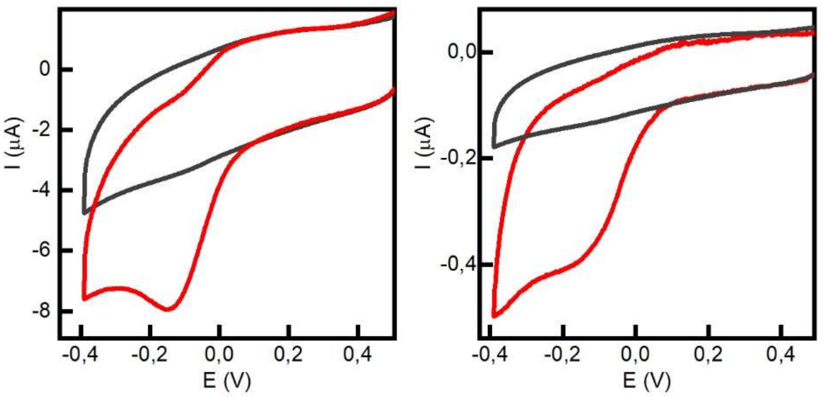

(a) (b)

Figure S1. Cyclic voltammetry experiments for (a) BsDyP and (b) PpDyP under aerobic conditions.

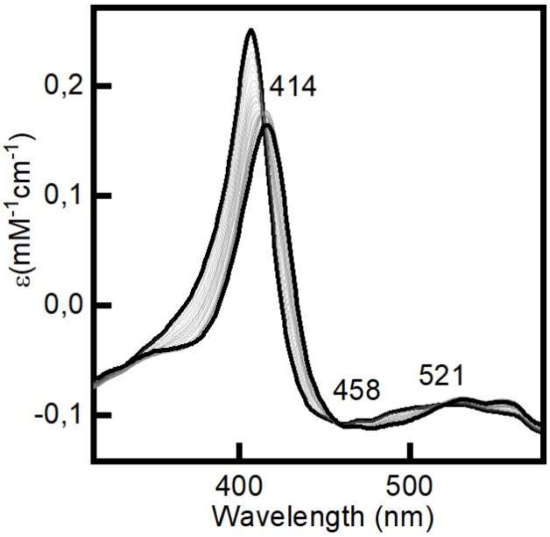

Figure S2. Conversion of BsDyP resting state to compound II species followed by UV-vis spectra. Isosbestic points can be identified at: 414 nm, 458 nm and 521 nm.

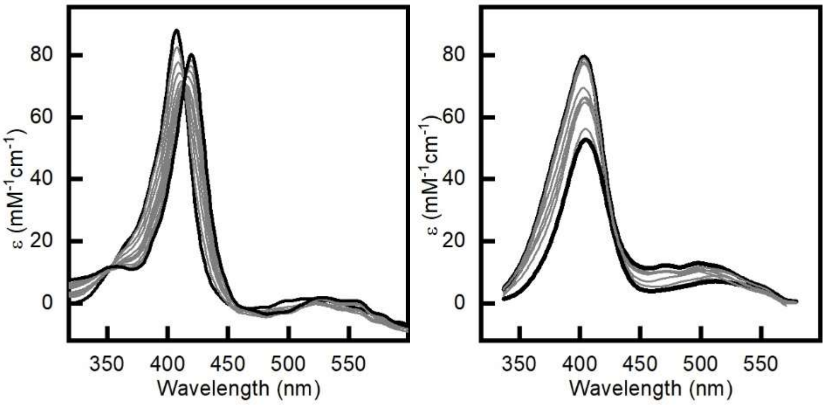

(a) (b)

Figure S3. Electrochemical titrations of (a) BsDyP and (b) PpDyP followed by UV-vis spectra. Both titrations start at 0.210 V and end at -0.190 V (thick solid lines).

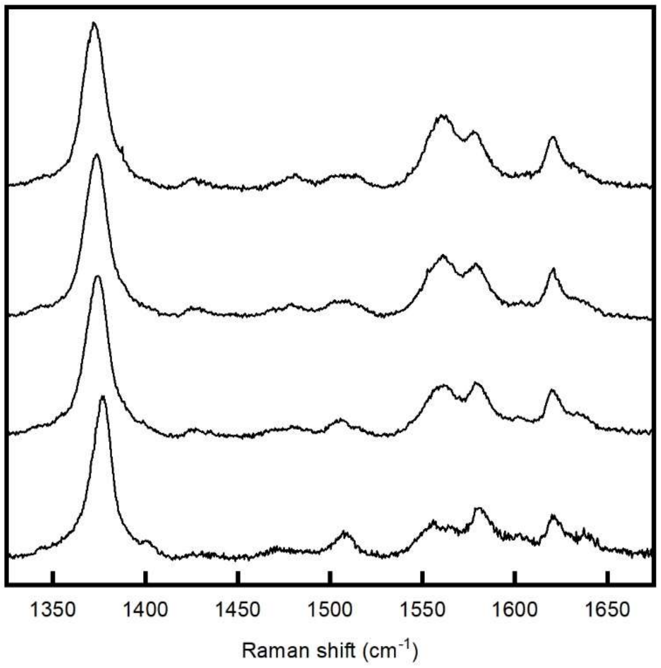

Figure S4. RR spectroscopy monitoring of BsDyP electrochemical titration to form compound II. From top to bottom: 0.260 V, 0.110 V, 0.040 V and -0.190 V applied potentials.

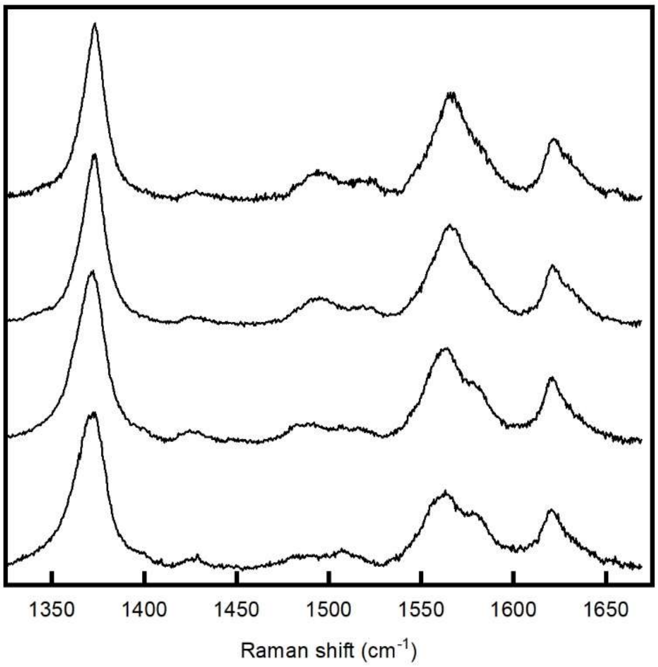

Figure S5. RR spectroscopy monitoring of PpDyP electrochemical titration to form compound II. From top to bottom: 0.260 V, 0.110 V, 0.040 V and -0.190 V applied potentials.

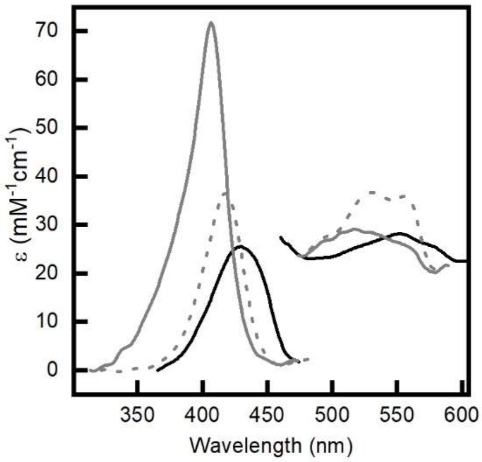

Figure S6. UV-vis absorption spectroscopy monitoring of BsDyP chemical titration with sodium dithionite. Resting state (thick grey line), compound II (dotted grey line), reduced form (thick black line).

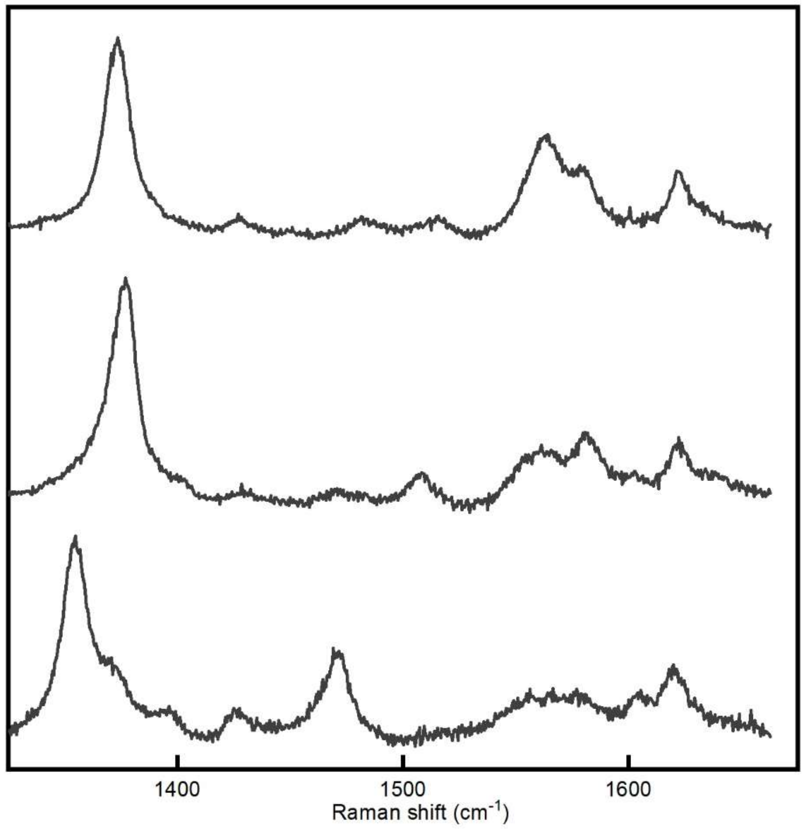

Figure S7. RR spectroscopy monitoring of BsDyP chemical titration with sodium dithionite. From top to bottom: resting state at 260 mV, first transition product: compound II (Soret at 418 nm) and second transition product: reduced form (Soret at 430 nm).

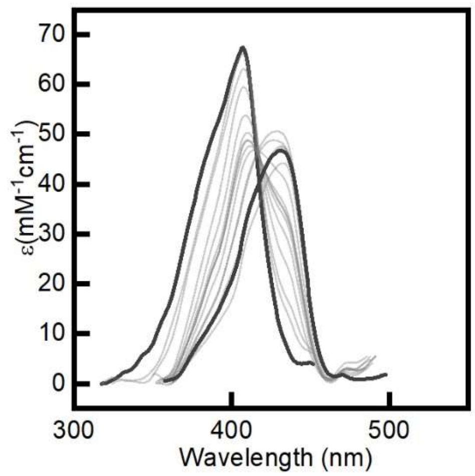

Figure S8. UV-vis absorption spectroscopy monitoring of PpDyP chemical titration with sodium dithionite. Soret maximum shifts from 405 nm to 431 nm (reduced form).

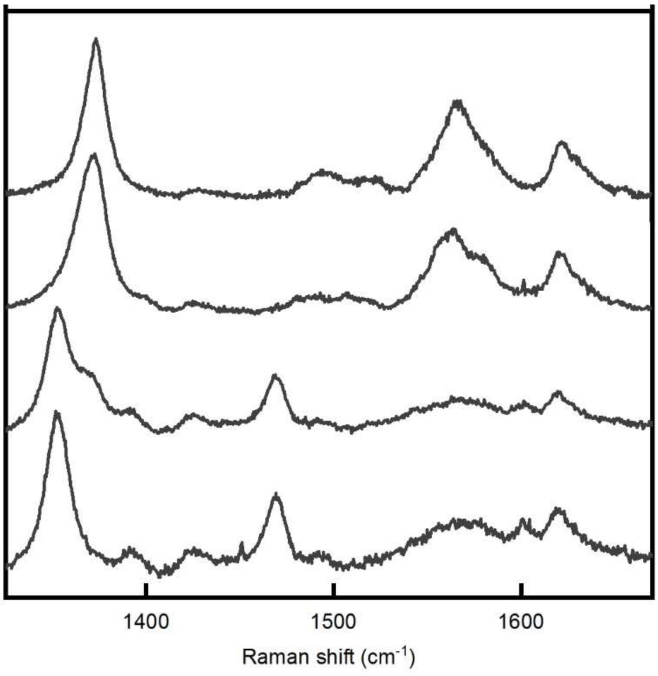

Figure S9. RR spectroscopy monitoring of PpDyP chemical titration with sodium dithionite. From top to bottom: resting state at 260 mV, first transition product: compound I, non-complete second transition and final product of second transition.

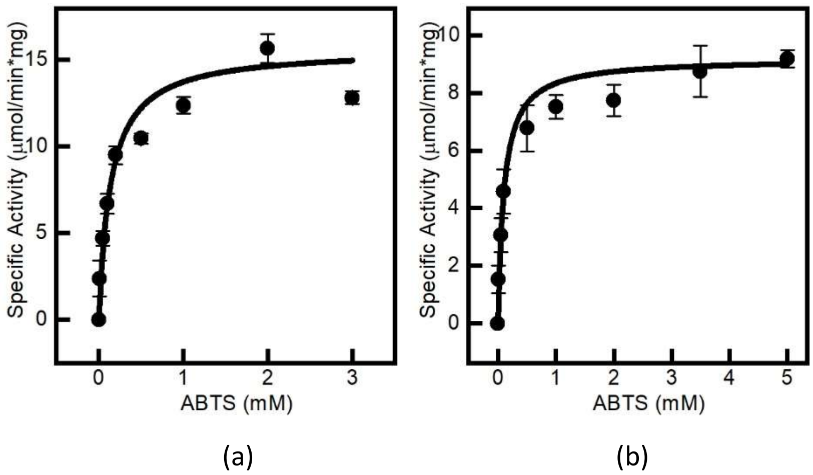

Figure S10. Steady state kinetic analysis for ABTS oxidation at pH 3.7 with electrochemical activation at poised potential -0.190 V for (a) BsDyP and (b) PpDyP.

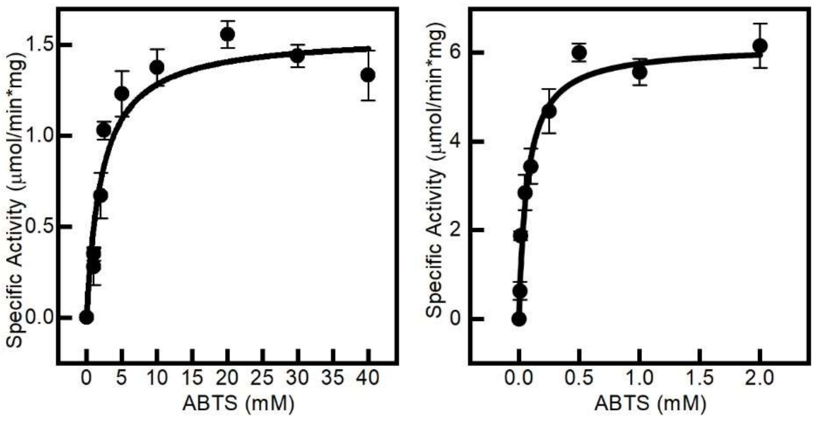

(a) (b)

Figure S11. Steady state kinetic analysis for ABTS oxidation at pH 8.5 with electrochemical activation at poised potential -0.190 V for (a) BsDyP and (b) PpDyP.

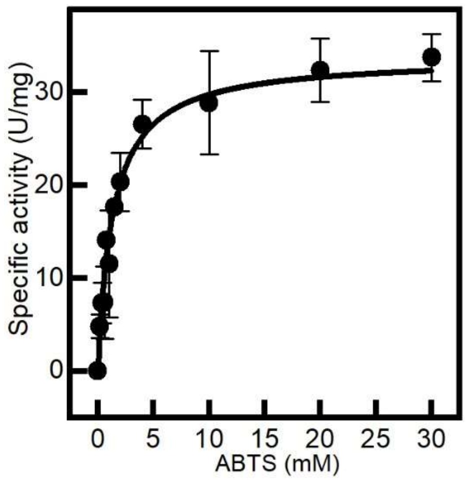

Figure S12. Steady state kinetic analysis for ABTS oxidation using H_2O_2 as electron acceptor for PpDyP at pH 4.3.

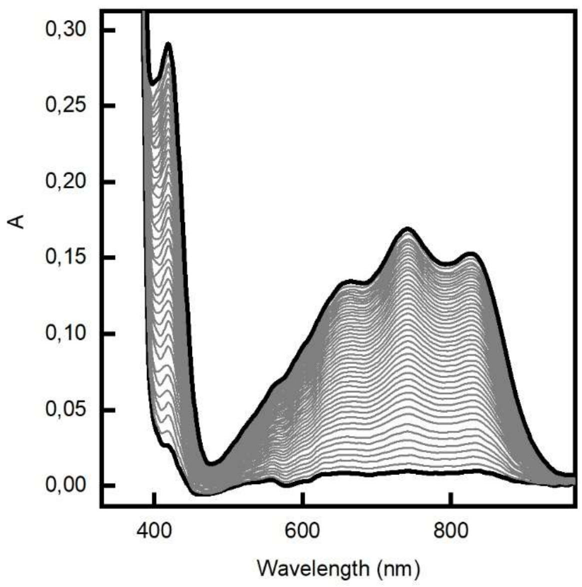

Figure S13. UV-vis absorption spectroscopy monitoring of ABTS oxidation, initialized at poised potential -0.190 V.
